# A rootstock-centered perspective on the regulation of alternate bearing in fruit trees

**DOI:** 10.1093/jxb/eraf460

**Published:** 2025-10-18

**Authors:** Pietro Carraro, Muhammad Yasir Naeem, Francesco Girardi, Alessandro Botton, Serena Varotto, Benedetto Ruperti, Claudio Bonghi

**Affiliations:** Department of Agronomy, Food, Natural resources, Animals and Environment, University of Padova, Viale dell’Università 16, 35020 Legnaro (PD), Italy; Department of Agronomy, Food, Natural resources, Animals and Environment, University of Padova, Viale dell’Università 16, 35020 Legnaro (PD), Italy; Department of Agronomy, Food, Natural resources, Animals and Environment, University of Padova, Viale dell’Università 16, 35020 Legnaro (PD), Italy; Department of Agronomy, Food, Natural resources, Animals and Environment, University of Padova, Viale dell’Università 16, 35020 Legnaro (PD), Italy; Department of Agronomy, Food, Natural resources, Animals and Environment, University of Padova, Viale dell’Università 16, 35020 Legnaro (PD), Italy; Department of Agronomy, Food, Natural resources, Animals and Environment, University of Padova, Viale dell’Università 16, 35020 Legnaro (PD), Italy; Department of Agronomy, Food, Natural resources, Animals and Environment, University of Padova, Viale dell’Università 16, 35020 Legnaro (PD), Italy; Max Planck Institute for Molecular Plant Physiology, Germany

**Keywords:** Carbohydrate allocation, developmental plasticity, epigenetic remodeling, floral transition, hormonal homeostasis, source–sink dynamics

## Abstract

Alternate bearing in most perennial fruit tree species refers to the phenomenon whereby high-yielding on-years are followed by low or nearly no fruiting off-years. This variability complicates orchard management, especially under unpredictable weather patterns. Alternate bearing is regulated by both endogenous and environmental signals, and recent studies suggest that rootstocks could play a role in its modulation. Beyond affecting scion growth and nutrient status, rootstocks influence developmental behavior through long-distance signaling. They participate in hormonal metabolism, nutrient uptake, water transport, and chromatin conformation in scion tissues. Epigenetic changes, including DNA methylation and histone marks, have been implicated in regulating flowering-related genes in response to environmental and developmental cues. This review explores possible contributions of the rootstock to alternate bearing through physiological, molecular, and epigenetic signals—such as signaling molecules and chromatin states associated with flowering—as working hypotheses. The role of rootstocks in shaping source–sink dynamics, interpreted throughout the resource budget model, and their potential influence on stress responses are also discussed in relation to alternate bearing rhythmicity. Finally, emerging strategies aimed at mitigating alternate bearing intensity, including genome editing, marker-assisted selection, and microbiome-based strategies, are highlighted as promising for stabilizing productivity under changing climate conditions.

## Teaser

This review explores how rootstocks affect alternate bearing through hormonal signaling, resource allocation, and epigenetic mechanisms, offering a framework to develop rootstock-centered breeding strategies for yield stabilization under climate stress.

## Introduction

**Fig. 1.**
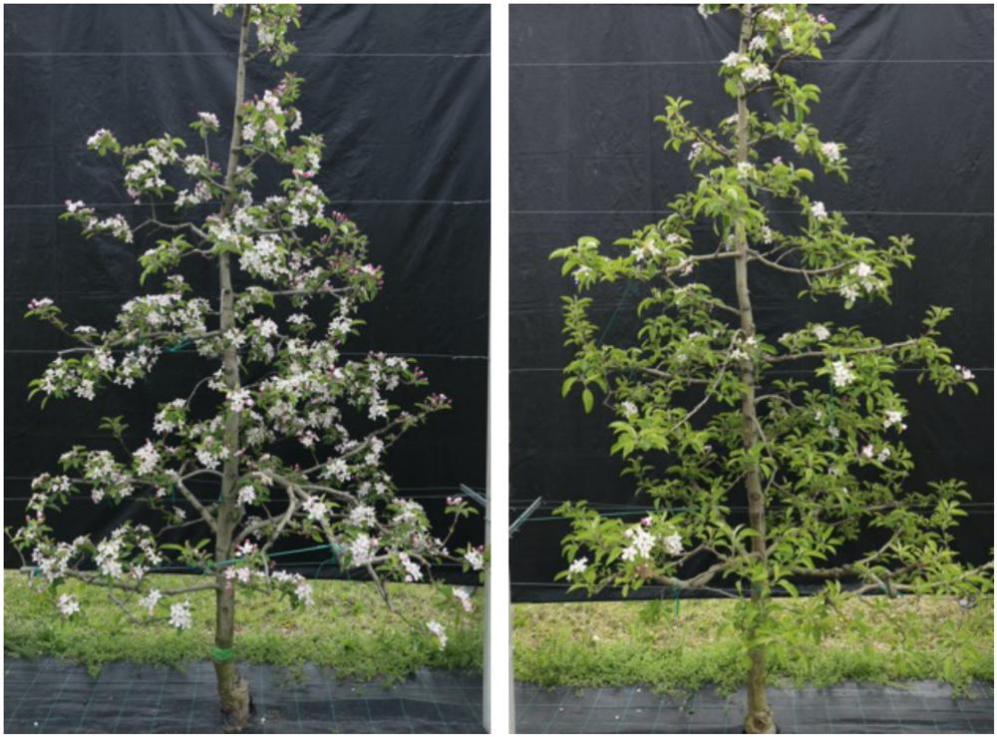
Effect of alternate bearing in grafted apple trees showing contrasting flowering behavior. Two genetically identical scions grafted onto different rootstocks and grown under the same environmental conditions. The tree on the left is in an on-year, characterized by abundant flowering, while the tree on the right displays an off-year, with markedly reduced floral density.

Perennial fruit trees experience alternate bearing (AB), a biennial pattern of fruit yields (Sharma *et al*., 2019), with on-years characterized at harvest by heavy crop load resulting in small-sized and poor quality fruits, and off-years, when fruit load is very scarce and cannot meet the production target of the grower (Fig. 1). This cyclical fruiting behavior has been observed in fruit tree species with nuts (hazelnuts, pecans, pistachios, and walnuts) and fleshy fruits (temperate: apples, apricots, pears, and prunes; subtropical: avocados, citrus, and olives; tropical: litchi and mango). Although often described as a biennial phenomenon, AB can also follow longer cycles, extending over 3 or even 4 years in some species and under certain environmental conditions (Monselise and Goldschmidt, 1982). This phenomenon creates challenges for reliable fruit production and agricultural planning, and its impact is increased in the scenario of a changing climate (Sharma *et al*., 2019). However, some perennial fruit crops, such as grapevine, are not very sensitive to AB, probably due to their indeterminate growth habit, the renewal of fruiting shoots each year, and their ability to buffer source–sink imbalances through canopy management (Martínez-Lüscher and Kurtural, 2021). In most perennial fruit trees, commercial orchards rely on grafted plants, where two genetically distinct individuals—the scion and the rootstock—function together as a single organism. The rootstock not only supports the scion physiologically but also modulates its growth, flowering behavior, and stress responses. Therefore, understanding how rootstock–scion interactions influence the AB events can provide new insights into the mechanisms underlying this phenomenon and inform more effective management strategies. In this context, this review re-examines the physiological, molecular, and epigenetic mechanisms underlying AB in temperate fruit tree species through the lens of rootstock–scion interactions, highlighting how rootstocks can modulate the balance of signals and resources that determine reproductive behavior.

## Mechanistic regulation of alternate bearing in tree fruit

AB results from a cyclical imbalance between vegetative and reproductive development, driven by a complex interplay between external environmental stimuli and internal regulatory networks under genetic control (Goldschmidt and Sadka, 2021). This imbalance manifests as an alternation in floral induction, which is suppressed during on-years and restored in off-years in response to the fruit load on the tree and environmental cues, mediated by hormonal and metabolic pathways. Two principal hypotheses, and not mutually exclusive, have been proposed to explain the cyclical suppression of floral induction underlying AB: one based on hormonal feedback, the other on carbohydrate depletion (Goldschmidt and Sadka, 2021; Campbell and Kalcsits, 2024). The hormonal hypothesis suggests that fruit-derived signals, particularly gibberellins (GAs), inhibit the transition of meristems to a reproductive state during on-years, whereas the carbohydrate-based hypothesis posits that the strong sink activity of developing fruits depletes carbohydrate reserves, particularly leaf starch (Alikhani-Koupaei and Aghdam, 2020), limiting the resources needed for floral induction. Building on these physiological insights, several crop load management strategies have been developed to mitigate AB by restoring balance between assimilate distribution and hormonal signaling across tree organs. These include the use of resting spurs, where selected fruiting spurs are intentionally left unharvested or unthinned in a given year to stabilize future flowering, as well as deblossoming and defruiting, which involve mechanical or chemical removal of flowers or young fruits to reduce the fruit load in on-years and promote more uniform yields over time (Monselise and Goldschmidt, 1982; Wünsche and Ferguson, 2005; Campbell and Kalcsits, 2024). These regulatory mechanisms ultimately affect the expression of key flowering genes—such as FLOWERING LOCUS T (FT), APETALA1 (AP1), TERMINAL FLOWER 1 (TFL1), and FLOWERING LOCUS C (FLC)—primarily in the scion organs (leaves, buds, and fruits). The developmental stages and regulatory pathways underlying floral induction are summarized in Box 1. In on-years, the high fruit and seed load is associated with suppression of FT and AP1 expression in leaves in citrus (Muñoz-Fambuena *et al*., 2011), and with increased TFL1-2 expression in buds in apple (Haberman *et al*., 2016), thereby inhibiting floral initiation for the following season. Conversely, in off-years, the reduced crop load alleviates this repression, allowing the resumption of FT and AP1 expression in leaves of citrus (Muñoz-Fambuena *et al*., 2011) and of TF expression in mango (Das *et al*., 2019), thus promoting the floral transition. Supporting these findings, Milyaev *et al*. (2021) showed that the reactivation of flowering-related genes in buds during off-years is associated not only with a decline in auxins but also with increased levels of thiamine, chlorogenic acid, and an adenine derivative, suggesting a broader metabolic reprogramming accompanying hormonal changes.

Box 1. **The flowering process in perennial fruit trees**

In angiosperms, flowering is a tightly regulated developmental event that arises from the transition of the shoot apical meristem from a vegetative to a reproductive identity. In perennial fruit trees, unlike in annuals, this remodeling is not deterministically associated with the juvenile-to-adult transition; it is a cyclical event that can recur every 2 years or more, influenced by endogenous and environmental cues. Flowering is usually divided into five developmental stages: induction, transition, initiation, differentiation, and anthesis (Samach and Smith, 2013). Floral induction and early stages of meristem re-programming happen during summer and autumn of the first year in temperate fruit crops, while anthesis (flower opening) happens the following spring. Floral induction is the first step and is mediated by a combination of exogenous signals (temperature, light, photoperiod, and water and nutrient availability) and endogenous cues (hormonal levels, nutritional status, and epigenetic marks (Blázquez *et al*., 2003; Tadayon and Hosseini, 2022). This pathway ends with the generation of florigenic signals, including the FT protein, which are produced in leaves and transported to the shoot apical meristems through the phloem (Azevedo *et al*., 2025). In perennial fruit trees, FT expression during floral induction has been observed in leaves of adult trees (e.g. Citrus: Muñoz-Fambuena *et al*., 2011), consistent with the Arabidopsis model. Expression in buds has also been documented (Shalom *et al*., 2012), and in some citrus cultivars, FT transcript levels can even be higher in buds than in leaves during the inductive period (Goldberg-Moeller *et al*., 2013; Haim *et al*., 2025b), suggesting species-specific or tissue-specific regulation of FT localization. A similar meristem-restricted expression pattern is observed in Narcissus (Noy-Porat *et al*., 2013), suggesting that species and developmental stage can influence FT transcript localization.

Unlike annuals, where flowering is often a qualitative and synchronous response, perennials exhibit a more plastic and quantitative flowering, wherein only a subset of meristems switch depending on resource availability and internal thresholds (Bangerth, 2009). Floral transition refers to the physiological and genetic reprogramming of the meristem upon induction, involving photoperiodic, vernalization, autonomous, and hormonal pathways regulating changes in gene expression (Mouradov *et al*., 2002; Madrid *et al*., 2021). This activates key floral identity genes, such as *APETALA1* (*AP1*), *LEAFY* (*LFY*), and *CAULIFLOWER* (*CAL*), and repression of *TERMINAL FLOWER1* (*TFL1*) (Wellmer *et al*., 2006; Zhu *et al*., 2020). The balance of these gene products determines whether the meristem will progress toward flower formation. Carbohydrates, including sucrose and trehalose-6-phosphate, have also been demonstrated to promote flowering in both model species and fruit crops (Wahl *et al*., 2013; Girardi *et al*., 2024).

Floral initiation marks the point of commitment to inflorescence development. Histological evidence indicates that meristems begin to develop and continue to grow in a floral shape, in a process that is highly cultivar and species dependent. In peach, floral initiation takes place in early July (Engin and Ünal, 2007). Similarly, in grapevine, inflorescence primordia appear during the summer: they are first detectable in *V. labrusca* and later recognized also in *V. vinifera* (Carmona *et al*., 2007). In the ABC(DE) model, differentiation of floral organs proceeds sequentially, with sepals being specified first, followed by petal, stamen, and carpel formation (Haughn and Somerville, 1988; Murai, 2013), and in many fruit species these organs are completely differentiated before dormancy in late autumn. Once the flowers have matured and buds develop, most temperate fruit trees enter into a period of rest during the winter, after which the flowers must open (anthesis) to allow pollination and fruit set in spring. Under this framework of staging, it becomes clear that the timing of internal–external signals that dictate reproductive development is a key control point for horticultural management.

Created with BioRender. Bonghi, C. (2025) https://BioRender.com/kz0oey2, and further edited in PowerPoint.

**Figure:**
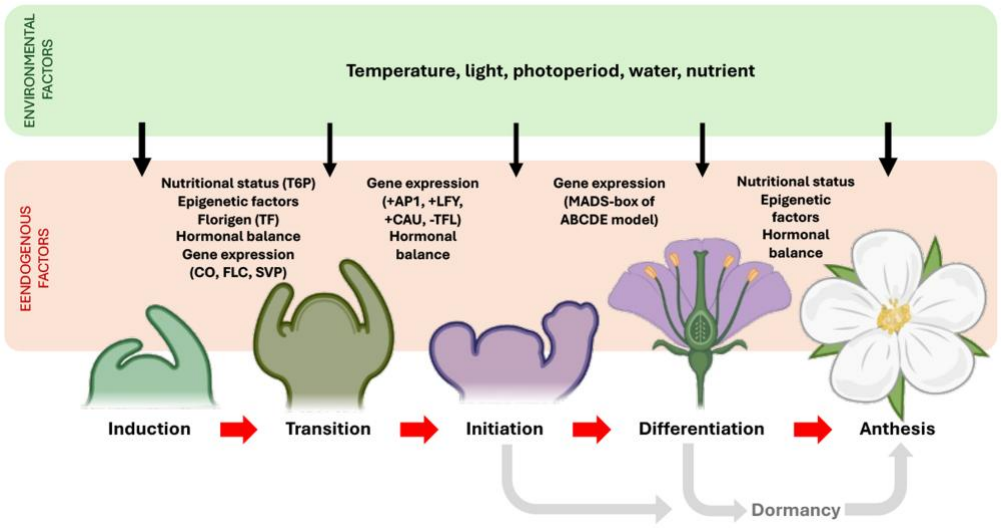

## Rootstock–scion interactions: a framework for new insights into alternate bearing

In grafted trees, the interaction between the genetically distinct rootstock and scion further amplifies the complexity of AB regulation. Although primarily adopted for combining desirable traits such as disease resistance, vigor control, and fruit quality, grafting also introduces a multilayered interaction in which the two partners communicate through the graft union (Goldschmidt, 2014). This communication involves more than the passive transport of water and minerals, encompassing an active and bidirectional exchange of signals that can shape tree development and productivity over time (see Box 2). Evidence from vegetables and model species indicates that mobile signaling molecules, particularly phytohormones and peptides, are well-established mediators of long-distance communication in grafted plants (Aloni *et al*., 2010; Pandita *et al*., 2023). Small RNAs and mRNAs have also been proposed to contribute to rootstock–scion interactions in grafted plants (Notaguchi and Okamoto, 2015), although recent findings urge caution in interpreting omics-based evidence due to potential sequencing artifacts and noise (Paajanen *et al*., 2025). While some studies have suggested that these molecules may cross the graft interface and influence gene expression, meristem behavior, and stress responses (Habibi *et al*., 2022), their systemic regulatory role remains to be fully validated.

Box 2. **Rootstock–scion communication in grafted fruit trees**

Grafted fruit trees are chimeric systems where the scion and rootstock, often of different genotypes or even species, interact through a complex network of signals exchanged across the graft union. This bidirectional communication is essential not only for vascular reconnection and structural integrity, but also for the long-term coordination of growth, development, and stress responses. Mobile molecules such as hormones, RNAs, proteins, and nutrients mediate these interactions, allowing the rootstock and scion to reciprocally influence each other’s physiology (Goldschmidt, 2014; Gautier *et al*., 2020).

Among the most studied forms of long-distance communication are phytohormones. Auxins, cytokinins (CKs), gibberellins (GAs), and abscisic acid (ABA) synthesized in the root system are translocated to the scion via the xylem, influencing apical dominance, shoot elongation, leaf senescence, and meristem activity (Warschefsky *et al*., 2016). For example, rootstock genotype can shape CK levels and thereby influence shoot branching patterns and vigor, as reported in apple and grapevine (Gautier *et al*., 2019).

In addition to hormones, mobile RNAs—including mRNAs, miRNAs, and siRNAs—have been proposed to move across the graft interface and regulate gene expression in the scion (Habibi *et al*., 2022). These molecules may influence transcriptional networks related to stress responses, development, and nutrient signaling. However, recent work by Paajanen *et al*. (2025) cautions that previous reports on long-distance RNA movement may have overestimated its functional relevance due to technical artifacts or contamination. This raises the need to reassess the extent to which mobile RNAs directly mediate cross-graft communication.

Despite these uncertainties, some siRNAs have been implicated in RNA-directed DNA methylation (RdDM), suggesting a possible epigenetic influence of the rootstock on the scion. This pathway, well characterized in model plants and observed in Citrus and grapevine, suggests that rootstock-derived small RNAs could guide epigenetic modifications across the graft union (Huang *et al*., 2021; Rubio *et al*., 2022; Martins and Law, 2023). These findings remain preliminary, and their functional significance in mature fruit trees is yet to be established.

Nutrient and water transport also represent key aspects of rootstock–scion communication. The rootstock determines root architecture, xylem vessel anatomy, and uptake efficiency, thereby affecting the uptake of nitrogen, phosphorus, potassium, and water. These factors are critical for photosynthetic performance and shoot development, and rootstocks with superior uptake traits have been shown to enhance scion growth and productivity in species such as apple and pistachio (Hayat *et al*., 2021; Mohammed and Hama Salih, 2024).

In parallel, the root microbiome, largely determined by the rootstock genotype, indirectly contributes to this communication. Recent studies performed in grapevine have demonstrated that both rootstock and scion genotypes shape the composition and function of root-associated microbiomes, with clear associations between microbial structure and phenotypic traits such as vigor, nutrient status, and stress adaptation (Lailheugue *et al*., 2024).

Altogether, the graft junction acts as a highly dynamic interface where chemical, molecular, and epigenetic signals converge. A more complete understanding of these processes in perennial fruit trees will be crucial to fully harnessing the potential of rootstocks as modulators of scion physiology and performance.

Created with BioRender. Bonghi, C. (2025) https://BioRender.com/rcoois4, and further edited in PowerPoint.

**Figure:**
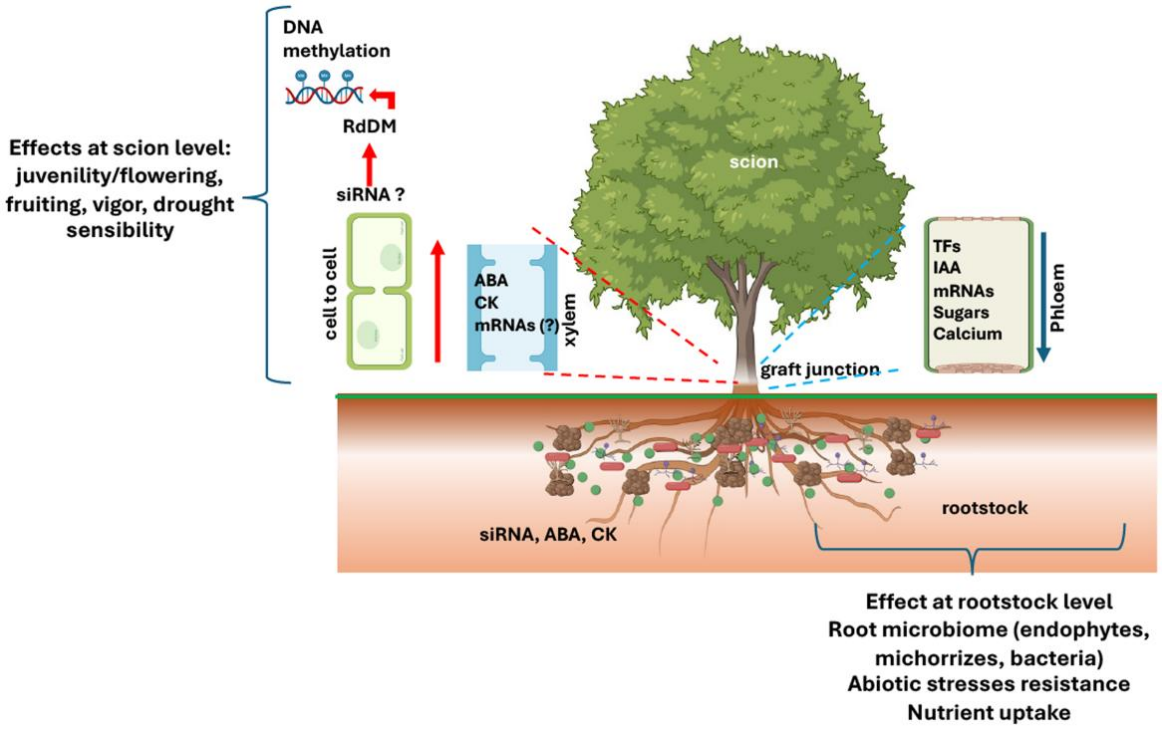

An increasing number of studies indicate that the rootstock is not a neutral partner, but rather an active modulator of the vegetative and reproductive behavior of the scion. In fruit trees, rootstock can influence the return bloom and yield regularity by altering hormone transport and sensitivity (Goldschmidt and Sadka, 2021), modifying water and nutrient uptake efficiency (Aguirre *et al*., 2001; Tworkoski and Fazio, 2011), and affecting gene expression profiles in the scion, including flowering-related genes as reported for FT expression in citrus and apple (Foster *et al*., 2014; Bennici *et al*., 2021). A large-scale analysis in apple cv. ‘Elstar’ involving >2000 trees (Krasniqi *et al*., 2013) further highlighted the importance of tree-to-tree variability in AB expression, proposing a conceptual model in which root-derived signals—including cytokinins (CKs), xylem-mediated transport of light and water signals, and the modulation of basipetal GA flow—contribute to the cyclical regulation of flowering through source–sink dynamics.

Recent evidence suggests that rootstock-induced effects may extend to chromatin-level modifications, potentially influencing epigenetic regulation in the scion; however, these observations remain preliminary and their consistency across woody species and developmental stages requires further clarifications (Warschefsky *et al*., 2016; Kapazoglou *et al*., 2021). Moreover, it should also be noted that most available evidence on graft-induced epigenetic and transcriptional modifications comes from studies on young or herbaceous plants under controlled conditions, and it remains unclear whether similar mechanisms operate in mature, field-grown fruit trees (Kapazoglou *et al*., 2021).

The outcome of rootstock–scion interactions often varies depending on the specific genetic combination and prevailing environmental conditions. Some rootstocks, by enhancing water transport or nutrient absorption, may promote vigorous vegetative growth by potentially hindering floral induction, through shifts in hormonal and carbohydrate balance. Under stress conditions, abscisic acid (ABA)-, CK-, and sugar-related signaling pathways—strongly influenced by the rootstock genotype—can play a decisive role in shaping the response of the plant and modulating the expression of AB (Li *et al*., 2017; Kviklys and Samuolienė, 2020).

In the following sections, we dissect the physiological, molecular, and epigenetic pathways through which rootstock–scion interactions regulate AB, emphasizing how rootstocks modulate the balance of signals and resources underlying reproductive behavior.

## Rootstock-mediated hormonal control of alternate bearing

Rootstocks influence hormonal pathways involved in flower induction, meristem identity, and bud development, all contributing to the extent of AB. The interplay between auxins, GAs, CKs, and ABA determines the switch from vegetative to reproductive growth, by modulating shoot apical meristem development and floral meristem identity in woody perennials (Khan *et al*., 2025). This hormonal balance is not fixed, but responds dynamically to factors such as fruit load, environmental stress, and the genetic background of both scion and rootstock (Goldschmidt and Sadka, 2021).

Rootstocks, in addition to the effects of fruit load, influence AB by modulating auxin dynamics, which in turn affect floral initiation. In apple, the cultivar ‘Honeycrisp’ displays variation in AB depending on the rootstock. In a comparative study using ‘Honeycrisp’ as the scion, auxin concentrations measured in the xylem of different rootstocks showed weak correlations with biennial bearing index, branch angle, and other growth-related variables (Lordan *et al*., 2017). Although correlative in nature, these findings suggest a potential role for rootstock-derived auxin dynamics in modulating floral induction and influencing AB in the scion.

GAs also play a major role in AB, particularly through their suppression of flowering during on-years. In mature apple trees, fruit-derived GAs such as GA_3_, and notably mixtures of GA_4_+GA_7_, suppress floral induction by up-regulating *MdTFL1-2* during the critical period after bloom, effectively reducing flowering in the next season (Haberman *et al*., 2016). Similarly, in olive, endogenous GA_3_ produced in the developing seeds suppresses floral induction in the following season, while exogenous GA_3_ applications around the pit-hardening stage similarly inhibit flower initiation (Zare *et al*., 2023). In pistachio, elevated levels of endogenous GAs in on-years correlate with reduced flowering in the subsequent season, though the specific isoforms have not been fully identified (Gündesli, 2020). In citrus, applications of exogenous GA_3_ during the floral differentiation period effectively reduce return bloom and are used commercially to mitigate excessive flowering and improve fruit set (Agustí *et al*., 2022; Haim *et al*., 2025a). In apple, trees grafted on dwarfing rootstock M9 showed higher GA levels and reduced flowering compared with those grafted on the semi-dwarfing B396 rootstock (Kviklys and Samuolienė, 2020). The inhibition of floral induction mediated by GA has been related to an increased expression of *MdTFL1-2*, a floral repressor homologous to Arabidopsis TFL1 (Haberman *et al*., 2016). In several species, DELLA proteins repress GA signaling and tend to accumulate in tissues with active GA synthesis, thereby reinforcing the inhibitory effect on the reproductive transition (Ito *et al*., 2018). They regulate plant flowering by both directly interacting with flowering-related genes (Ito *et al*., 2018) and indirectly modulating GA levels, as demonstrated by the overexpression of MiSLR1 and MiSLR2—two DELLA genes from mango—in transgenic Arabidopsis, which resulted in early flowering and up-regulation of flower-related gene expression (Yang *et al*., 2024).

In Arabidopsis, CKs generally act as positive regulators of floral induction and bud outgrowth, often antagonizing the inhibitory effects of GAs (D’Aloia *et al*., 2011). This suggests that, in fruit trees, rootstocks that promote CK biosynthesis or facilitate CK translocation to the scion might help support return bloom and reduce AB expression. In apple, differences among rootstocks in CK signaling and sugar metabolism have been linked to variations in biennial bearing, with genotypes exhibiting higher CK activity and more balanced crop load showing reduced AB expression and improved flowering regularity (Kviklys and Samuolienė, 2020). The balance between auxin and CKs seems to play a central role in bud fate, but the threshold at which this ratio triggers floral induction varies depending on genotype and environmental context (Lordan *et al*., 2017). In line with this, treatments aimed at modulating the GA/CK balance—particularly those enhancing CK activity—have been applied in apple, improving floral consistency and mitigating AB (Ramírez *et al*., 2004).

ABA, typically involved in stress responses and bud dormancy, is also implicated in the regulation of flowering. In particular, Shu *et al*. (2018) observed that, under suboptimal conditions, ABA modulates floral induction, partly by antagonizing GA activity and alleviating its inhibitory effect on flowering. In Citrus, ABA levels in buds increase as GA concentrations decline during the floral transition, further supporting a possible antagonistic interaction between the two hormones (Shalom *et al*., 2014).

Besides hormone biosynthesis, rootstocks can affect hormone action by modifying long-distance transport and the sensitivity of scion tissues to hormonal signals. In apple, dwarfing rootstocks such as M9 limit shoot growth and developmental transitions by disrupting hormone signaling and sugar allocation. Down-regulation of auxin influx transporters (MdAUX1 and MdLAX2) and flavonoid overaccumulation are proposed to reduce basipetal auxin transport, leading to altered shoot architecture and growth termination (Foster *et al*., 2017). Such modifications can reshape local hormonal gradients and influence the feedback mechanisms that regulate meristem activity, bud reactivation, and the timing of floral induction. Further evidence for the role of hormonal transport in floral regulation is provided by Haim *et al*. (2020), who demonstrated that auxin exported from developing fruits can suppress flower induction in distal buds of citrus and olive, independently of local carbohydrate or ABA levels. This supports the view that modulating auxin fluxes—possibly under rootstock control—may influence meristem differentiation, as demonstrated by Shi *et al*. (2018) in *Arabidopsis thaliana*, and contribute to AB behavior. Consistent with this model, field observations in apple have shown that rootstocks limiting auxin transport to the shoot apex are associated with a more stable return bloom in the subsequent season (Lordan *et al*., 2017). In pistachio, genotypic variation in auxin transport and CK movement between root and shoot has been linked to differences in floral initiation and the severity of AB (Mohammed and Hama Salih, 2024).

Overall, the available evidence suggests that rootstocks can modulate hormone biosynthesis, transport, and sensitivity in the scion, potentially influencing gene expression and meristem development relevant to AB.

## Rootstock influence on source–sink dynamics in alternate bearing regulation

AB in perennial fruit trees has traditionally been interpreted as a consequence of internal competition for limited carbon and nutrient resources. When reproductive demand is high, carbohydrates and nutrients are preferentially allocated to developing fruits, limiting their availability for vegetative growth and floral initiation in the following season. This source–sink paradigm underpins conceptual models such as the resource budget model (RBM), which formalizes the energy balance of the tree as the difference between photosynthetic gain and reproductive cost (Isagi *et al*., 1997), and the biennial bearing index developed in apple to quantify tree-level AB tendency and link it to physiological factors (Krasniqi *et al*., 2013). A more recent extension of the RBM, developed by Esmaeili *et al*. (2021), incorporates both the finite storage capacity of tree reserve and density-dependent accumulation dynamics. By removing the rigid threshold of reproduction assumed in the classical RBM, this refinement allows the model to capture species-specific yield patterns—including low but non-zero production in off-years. Moreover, it provides a more flexible and realistic framework for describing AB dynamics and their synchronization. However, this model does not explicitly account for grafted trees and the potential influence of rootstock–scion interactions on resource allocation and yield patterns. Heavy crop load has been shown to deplete non-structural carbohydrate (NSC) reserves in roots, stems, and shoots, while lighter crop loads allow NSC replenishment, supporting floral transition and return bloom. During fruit development, starch is converted into soluble sugars—mainly sucrose, glucose, and fructose—which are preferentially allocated to fruits. This diversion of assimilates impairs bud differentiation and reinforces AB expression (Rossouw *et al*., 2024). Nitrogen (N) and potassium (K) follow a similar pattern, being allocated to fruits during on-years and accumulating in vegetative tissues during off-years (Rosecrance *et al*., 1996). However, these trends may not apply uniformly across species: in olive, carbohydrates appear less tightly coupled to AB intensity, underscoring the need for species-specific interpretation (Bustan *et al*., 2011).

Specific examples further highlight how these physiological mechanisms manifest in individual species. In mango, for instance, Capelli *et al*. (2021) documented how heavy fruit load inhibits vegetative bud outgrowth and depletes starch in non-fruiting axes, delaying floral development through auxin-mediated pathways. Consistently, Rossouw et al. (2024) showed that fruit thinning allows NSC recovery in shoots and buds, enhancing floral induction and mitigating AB intensity. Recently, Campbell and Kalcsits (2024) reviewed biennial bearing control in apple, highlighting how rootstocks may alter carbohydrate reserve allocation and modulate AB severity. Similar cause–effect relationships have been proposed in mango, where rootstock-induced differences in carbohydrate partitioning affected scion carbohydrate status and, consequently, the development of AB behavior (Vittal *et al*., 2023). Similarly, in mandarin, rootstock choice influenced the carbohydrate status of buds and leaves and modified return bloom, supporting the hypothesis that rootstock-mediated resource allocation contributes to AB regulation (Stander *et al*., 2018).

The complexity of source–sink dynamics has been explored using process-based models such as CLM5-FruitTree, which integrates environmental, physiological, and management variables to simulate yield response (Dombrowski *et al*., 2022). While these models do not explicitly simulate AB, they demonstrate how management interventions interact with genotype-dependent source–sink relationships and influence yield stability. In this context, the capacity of a given rootstock–scion combination to buffer resource imbalances and maintain bud viability becomes crucial, and rootstock choice may enhance the effectiveness of crop load management practices (Wünsche and Ferguson, 2005; Campbell and Kalcsits, 2024). For instance, in ‘Valencia’ orange, Lo Bianco *et al*. (2018) showed that long-term deficit irrigation strategies—including partial root zone drying—did not significantly modify the AB index over multiple seasons, suggesting that water availability alone may not over-ride the endogenous regulatory circuits of AB in this cultivar.

## Mineral nutrition and water relations mediated by rootstocks in alternate bearing

Mineral nutrition influences the source–sink balance in fruit trees and it is tightly modulated by the rootstock and its interaction with the scion, affecting nutrient uptake and distribution between vegetative and reproductive organs, and overall tree vigor (Zuazo *et al*., 2006; Ikinci *et al*., 2014; Valverdi *et al*., 2019; Mohammed and Hama Salih, 2024). However, direct and consistent evidence linking these nutritional effects to AB remains limited and sometimes contradictory. In apple, rootstock vigor alone does not reliably predict bearing consistency. Kviklys *et al*. (2016) found no consistent correlation between the vigor class of semi-dwarf, dwarf, and super-dwarf rootstocks and AB consistency. Although some dwarf and super-dwarf rootstocks may improve yield stability in certain apple varieties, crop load remains the strongest predictor of AB, irrespective of rootstock type (Kviklys and Samuolienė, 2020). Conversely, Valverdi *et al*. (2019) reported that dwarfing apple rootstocks, characterized by higher N and lower K uptake, are associated with reduced AB compared with more vigorous rootstocks. Moreover, the scion genotype, alongside crop load, significantly affects the allocation of mineral and carbon resources within the tree (Wünsche and Ferguson, 2005; Atay and Atay, 2024), contributing to AB through their coordinated effects.

In pistachio, in contrast to apple, more vigorous rootstocks have been associated with lower AB index, probably due to their improved nutrient uptake, increased carbohydrate reserves, and better resilience to stressors (Akbari *et al*., 2024). These findings highlighted the influence of species-specific strategies on how rootstock traits modulate AB behavior.

Reserve depletion during the on-year plays a crucial role in regulating AB, influencing nutrient allocation within the plant in tandem with growth of the scion. Deficiencies in specific nutrients—particularly in K—may exacerbate AB patterns by impairing flower initiation, fruit set, and vegetative recovery (Rosecrance *et al*., 1998; Roversi and Ughini, 2006).

Rootstocks modulate scion nutrient uptake and partitioning, with studies reporting significant variability in leaf N, P, and K concentrations across different apple rootstock–scion combinations (Fallahi *et al*., 1984; Aguirre *et al*., 2001). Less vigorous rootstocks may sustain higher N and higher photosynthesis rates under low-N conditions, partly through shifts in rhizosphere microbial communities (Chai *et al*., 2019). Root-induced modulation of the rhizosphere microbiome, together with root architecture and hydraulic properties, is increasingly recognized as a key factor shaping nutrient uptake efficiency in the scion (Bhattacharyya and Jha, 2012; Castellano-Hinojosa *et al*., 2023). While the connection between microbiome-mediated nutrient dynamics and AB remains speculative, its potential contribution to bud development and return bloom warrants further investigation.

Water absorption is also fundamental for mineral nutrition, with rootstock hydraulic conductance playing a major role in managing water and nutrient delivery to the scion (Brar *et al*., 2022). In several fruit species, size-controlling rootstocks often limit hydraulic and mineral supply due to narrower xylem vessels, thus constraining vigor and yield (Hayat *et al*., 2021). Positive correlations between the xylem vessel anatomy and hydraulic conductance have been linked to tree vigor in pear and apple (Atkinson *et al*., 2003; reviewed by Hayat *et al*., 2021), although the scion carbohydrate storage capacity appears to remain the dominant determinant of AB (Jupa *et al*., 2021).

Water status, nutrient availability, and hormonal signaling collectively transmit environmental and internal cues to developing meristems, thereby influencing bud fate and the resulting AB patterns. Notably, positive correlations between glucose, auxin, and ABA in buds of apple trees suggest that sugar metabolism and hormonal signaling interact synergistically during flower induction, with rootstock-mediated effects on these pathways influencing AB (Kviklys and Samuolienė, 2020).

## Epigenetic regulation of alternate bearing through rootstock–scion interactions

Epigenetic mechanisms—including DNA methylation, histone modifications, and non-coding RNA activity—are increasingly recognized as regulatory layers that modulate gene expression and chromatin architecture, without altering the underlying DNA sequence, in response to environmental and developmental cues (Hou *et al*., 2022). These processes contribute to transcriptional plasticity, allowing perennial plants to adjust flowering patterns across seasons, a hallmark of AB. At the molecular level, epigenetic control in plants is exerted through DNA methylation, post-translational histone modifications, and regulatory non-coding RNAs, which collectively establish and maintain specific chromatin states. DNA methylation patterns are written, maintained, or erased by distinct families of methyltransferases and demethylases, often guided by siRNAs via the RNA-directed DNA methylation (RdDM) pathway (Martins and Law, 2023). Histone modifications—including acetylation and methylation at key residues—contribute to the activation or repression of gene expression by altering chromatin accessibility, with marks such as H3K4me3 and H3K27me3 serving as canonical indicators of active or repressed transcription, respectively (Le *et al*., 2025). These mechanisms confer chromatin plasticity and memory, allowing plants to reprogram their development in response to internal and external cues.

For a comprehensive overview of plant epigenetic regulatory mechanisms, the reader is referred to Martins and Law (2023) and Le *et al*. (2025).

In this review, we focus on how these epigenetic processes intersect with the hormonal, nutritional, and developmental factors that underlie AB in fruit trees, and on the emerging evidence for rootstock-mediated effects on the epigenetic landscape of the scion. While much of what is known comes from model plants such as Arabidopsis, several studies in fruit trees—particularly in Citrus—have begun to elucidate how epigenetic modifications at specific flowering regulators, such as CcMADS19, contribute to the biennial bearing pattern (Agustí *et al*., 2020; Mesejo *et al*., 2022). Fruit presence in *Citrus clementina* is associated with enrichment in an active transcription mark, namely H3K4me3, in the *CcMADS19* locus in the neighboring leaves. The expression of *CcMADS19* represses the Citrus FT ortholog during the floral induction period (Agustí *et al*., 2020) and inhibits flower differentiation when fruits are present. However, Mesejo *et al*. (2022) observed that in young, but not old, leaves *CcMADS19* is silenced and enriched in repressive H3K27me3 marks, indicating that this silenced version of the floral repressor can be mitotically transmitted to the newly emerging leaves in axillary buds for inducing flowering. These observations suggested that in Citrus, resetting of the chromatin state at the *CcMADS19* locus and vegetative sprouting are necessary for the new leaves to respond to floral inductive signals (Mesejo *et al*., 2022). Given that the growing fruit represses lateral bud outgrowth, the renewal of the vegetative shoots mainly occurs after the end of the on-years, with harvest, therefore giving rise to biennial bearing. The horticultural consequence of the latter is that promoting vegetative growth (for instance, by mechanical pruning) mitigates yield alternation in Citrus (Mesejo *et al*., 2020).

Beyond Citrus, studies in Arabidopsis (Rudolf *et al*., 2024) and other model plants provide mechanistic insight into how epigenetic regulators interact with hormonal pathways central to AB. While these mechanisms remain to be directly demonstrated in perennial fruit trees, they offer valuable hypotheses for exploring how rootstock–scion interactions might influence flowering behavior through chromatin-level regulation. In Arabidopsis, genes involved in auxin biosynthesis—such as YUCCA (YUC), CYTOCHROME P450 (CYP), and TRYPTOPHAN AMINOTRANSFERASE 1/TRYPTOPHAN AMINOTRANSFERASE-RELATED (TAA1/TAR)—are regulated by H3K27me3 and H2A ubiquitination, while histone demethylases targeting H3K4me3 can further modulate their expression (Cui *et al*., 2021; Solanki and Shukla, 2023). Auxin transporters such as PINs are also regulated by changing levels of H3K27me3 during leaf differentiation (Lafos *et al*., 2011; He *et al*., 2012). Histone acetyltransferases (HATs) enhance transcription of auxin-responsive genes such as auxin-responsive factors (ARFs), while histone deacetylases (HDACs) suppress them, adjusting auxin sensitivity to environmental stimuli (Sakamoto *et al*., 2022; Liu *et al*., 2024). These pathways may contribute to temporal fluctuations of auxin observed between on- and off-years. Current evidence in grafted trees is mainly correlative; studies in apple indicate that rootstocks can modulate auxin metabolism and transport in scion, which may contribute to changes in shoot architecture and potentially influence floral development (Foster *et al*., 2017; Lordan *et al*., 2017). In addition, co-regulation of auxin-related mRNAs by trans-acting siRNAs (tasiRNAs) and miRNAs (Sunkar and Zhu, 2004) raises the possibility that auxin signaling in the scion might be modulated by epigenetic interactions at the graft interface, although such mechanisms are still debated (Habibi *et al*., 2022; Paajanen *et al*., 2025).

In fruit trees, GAs can affect AB through their action in floral and fruit development, and on biomass (Girardi *et al*, 2024). At the epigenetic level, high GA levels are associated with chromatin decondensation and a reduction in global DNA methylation (Manoharlal *et al*., 2018). Chromatin-remodeling complexes (CRCs), such as SWI/SNF subunits BRM, SWI3C, and INO80, positively regulate GA biosynthesis genes (Sarnowska *et al*., 2013; Li *et al*., 2018). DELLA proteins have been proposed as hubs integrating hormonal and epigenetic signaling by interacting with CRC components such as BRM, SWI3C, and PKL (Sarnowska *et al*., 2013; Y. Zhang *et al*., 2023), and histone (Huang *et al*., 2023), thereby influencing chromatin architecture and gene expression. Since the rootstock can modulate the GA level in the scion—as observed in apple, where dwarfing rootstocks are associated with higher GA accumulation and reduced flowering (Kviklys and Samuolienė, 2020), DELLA-mediated pathways may represent an important node of hormonal and epigenetic crosstalk.

Evidence from Arabidopsis and *Vitis vinifera* indicates that CK biosynthesis and metabolism are subjected to epigenetic regulation. In Arabidopsis, the CRC SWI/SNF-associated protein 73B negatively regulates CK biosynthesis (Jégu *et al*., 2015) while, in *V. vinifera*, two siRNAs (id4 and id65) target CK metabolic genes, including the AtIPT3 ortholog (Carra *et al*., 2009). Further insights from Arabidopsis show that chromatin remodeling contributes to CK-mediated control of shoot meristem identity: at the WUSCHEL (WUS) locus, CKs facilitate the loss of the histone mark H3K27me3 during shoot regeneration (Zhang *et al*., 2017). A similar mechanism has been observed in axillary buds (Wang *et al*., 2017), reinforcing the idea that CK-driven chromatin states contribute to meristem plasticity. Together, these findings support the hypothesis that epigenetic control of CK pathways may modulate vegetative growth and, by extension, influence AB tendencies in fruit trees.

Nutrient status may also shape chromatin states relevant to AB. Genes involved in nitrogen acquisition (e.g. *NRT1.1*, *NIA1*, and *AMT* genes) and phosphate transport (e.g. *PHT* genes) are known to be regulated epigenetically through DNA methylation, histone modifications, and small RNAs (Kumar *et al*., 2022; H. Zhang *et al*., 2023). Although similar studies in fruit trees are limited, nutrient fluctuations occurring between on- and off-years may plausibly trigger chromatin remodeling, thereby influencing flowering potential and growth rhythms. Since the rootstocks differ in their capacity for nutrient uptake and allocation, graft combination might modulate these epigenetic effects, although current evidence remains preliminary and speculative (Kapazoglou *et al*., 2021).

Water availability, particularly under drought stress, is another factor recognized to induce genome-wide epigenetic reprogramming. In Arabidopsis, the histone demethylase JMJ17 regulates dehydration stress-responsive genes through histone modifications, with involvement in the activation of genes such as *DREB* and *LEA* playing a key role in the stress response (Huang *et al*., 2019). In fruit trees, rootstocks with distinct water uptake capacities may influence these physiological and epigenetic responses in the scion, potentially contributing to seasonal variation in AB expression.

These observations suggest that the influence of rootstocks on AB may result from a multilayered integration of chromatin remodeling, hormonal dynamics, nutrient availability, and stress responses, all converging at the rootstock–scion interface. A deeper understanding of how these layers interact, particularly through epigenetic regulation, may help clarify the mechanisms underlying yield instability in fruit trees.

## Towards the development of new rootstocks to control AB

**Fig. 2.**
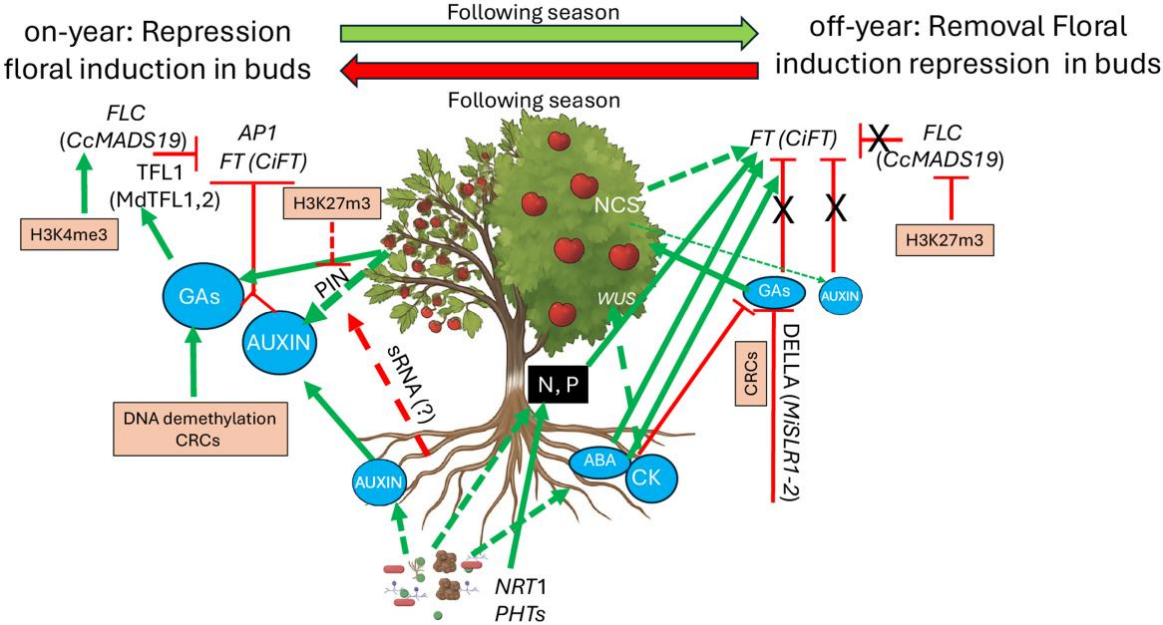
Mechanisms governing alternate bearing in grafted fruit trees: hormonal, epigenetic, nutrient, and the microbiome. This diagram provides an in-depth overview of the complex interplay between hormonal signaling, epigenetic modifications, nutrient dynamics, and microbiome influence on AB expression in grafted fruit trees: In on-years, developing fruit can translocate auxins towards the buds, supplied also by the rootstock, as well as gibberellins (GAs). Auxin translocation is mediated by auxin efflux carriers (PINs), which may be epigenetically regulated by sRNA (root-siRNA, although this aspect is still debated) and by the repressive histone mark (H3K27me3) as observed in model species. GA biosynthesis may also be modulated by DNA demethylation and chromatin-remodeling complexes (CRCs). The accumulation of these two hormones stimulates the expression of *TFL1* homologs (e.g. *MdTFL1* and *MdTFL2* in apple), repressing key flowering induction genes such as *AP1* and *FT*, as well as their homologs in fruit trees (e.g. *CiFT*). The permissive histone mark H3K4me3 has been associated with activation of floral repressors such as FLC homologs (e.g. *CsMADS19)*, which repress *CiFT.* In off-years, the reduction in fruit-derived auxins and GAs alleviates their inhibitory effects, allowing the activation of *CiFT*, which promotes floral induction in the buds. In Arabidopsis, it has been observed that the shift toward vegetative growth and increased CK signaling supports WUS expression *de novo* in the leaf axil to promote axillary meristem initiation. Repressive marks such as H3K27me3 may silence floral repressor genes such as *CsMADS19*, and DELLA proteins (e.g. *MiSRL1* and *2*, in mango) may interact with CRC to further relieve GA-mediated flowering repression. Variations in ABA sensitivity, may enhance the expression of flowering inducers, contributing to the floral transition. Finally, reduced crop load during off-years facilitates mobilization of non-structural carbohydrates (NSCs) and water, supporting floral induction. In both on- and off-years, rootstocks influence the rhizosphere microbiome, which modulates nutrient availability and phytohormone dynamics (particularly the ABA content), resulting (although this aspect is still under investigation) in a positive influence on flower induction, particularly during stressful periods. T-shaped lines indicate repression; green arrows indicate a positive effect. Solid and dashed lines represent mechanisms activated in fruit trees or in plant models only, respectively. X depicts the removal of repressor action. The thickness of arrows represents the level of transportation toward target organs. Created with BioRender. Bonghi, C. (2025) https://BioRender.com/wplz92y, and further edited in PowerPoint.

Understanding the complex factors that govern AB is essential for stabilizing fruit production. As discussed in previous sections, AB is influenced by hormonal signaling, nutrient allocation, and epigenetic regulation. A visual overview of the key processes involved in AB is provided in Fig. 2, offering mechanistic insights for developing breeding programs that target rootstock traits to mitigate AB expression in the field. These efforts focus on physiological attributes such as hormonal adjustment, carbon storage capacity, and abiotic stress tolerance to identify genotypes that buffer AB effects. Evidence from pistachio breeding programs, for example, has shown that genotypic differences in bearing behavior are sufficiently heritable to support the selection of low-AB cultivars or rootstock–scion combinations (Kallsen *et al*., 2007). Molecular strategies—especially marker-assisted selection and genome-wide association studies—have facilitated the identification of quantitative trait loci associated with AB in scions (Guitton *et al*., 2012), and traits relevant to the rootstock-mediated control of AB (Hayat *et al*., 2025). In apple and peach, innovations in high-throughput phenotyping—particularly thermal imaging and spectral sensing—have enabled the precise evaluation of physiological parameters such as stress tolerance, water status, and canopy health (Coupel-Ledru *et al*., 2019; Hayat *et al*., 2025). These advancements have facilitated the analysis of the factors influencing yield stability under variable environmental conditions, supporting the development of more resilient cultivars (Coupel-Ledru *et al*., 2019). High-throughput approaches enabled by these technologies facilitate rapid screening of large genotype collections and have been instrumental in isolating rootstocks that modulate stress responses during on- versus off-years. In Citrus, for example, spectral data have been used to assess canopy chlorophyll and water status, which facilitates screening of rootstocks with enhanced drought-buffering ability (Kuzhiyil *et al*., 2025). These strategies have produced promising results, but more experimental approaches—genome editing of hormonal and epigenetic regulators, or rootstock selection based on microbiome-enhancing traits—are under development. This set of studies opened the door to modulating genes involved in the control of flowering, hormone biosynthesis, and chromatin remodeling using CRISPR/Cas9 technology, which could enable rootstock-specific AB regulation (Hodaei and Werbrouck, 2023). As in peach, preliminary genome editing experiments have demonstrated the potential of targeting flowering pathway genes, such as *PpTFL1*, to influence floral induction timing (Windham, 2020; reviewed in Hayat *et al*., 2025). To translate these complex findings into practical applications, a more integrated approach is required, one that combines multi-seasonal field trials with remote sensing technologies and environmental monitoring systems to monitor and optimize rootstock performance under varying environmental conditions, as demonstrated in Citrus rootstock evaluation (Ampatzidis *et al*., 2019).

Recent studies in grapevine (Harris *et al*., 2023) and avocado (Núñez-Lillo *et al*., 2024) have demonstrated that gene expression profiles in the scion are shaped by both rootstock genotype and environmental factors, such as temperature and solar radiation. By integrating transcriptomic analyses with climatic data, these approaches enable the identification of genotypes with enhanced buffering capacity and adaptive plasticity. Such integrative frameworks are increasingly valuable for selecting rootstocks capable of mitigating AB and sustaining yield stability in variable growing environments.

## Conclusions and future perspectives

In the face of increasing climatic instability—characterized by erratic precipitation patterns, rising temperatures, and expanding periods of abiotic stress—AB is expected to intensify in many perennial fruit crops. Within this scenario, the functional role of the rootstock extends beyond structural support or vigor modulation, encompassing a complex regulatory influence on processes that govern the temporal coordination between vegetative growth and reproductive effort. Indeed, the genetic nature of rootstocks, which can belong to the same species, different species, or be interspecific hybrids—such as those used in apple (Marini and Fazio, 2018) and *Prunus* species (Ling *et al*., 2025)—plays a fundamental role in influencing hormonal homeostasis, carbohydrate partitioning, nutrient uptake dynamics, gene expression regulation, and, increasingly, interactions with the root-associated microbiome, as discussed in the previous sections.

Breeding efforts aiming to reduce AB incidence must therefore prioritize physiological and molecular traits that confer resilience across fluctuating environmental conditions. Rootstocks capable of sustaining stable hormone signaling—particularly balancing auxin, GA, CK, and ABA pathways—under varying crop loads and stress intensities are of particular interest. Genotypes that support efficient carbon storage during off-years and avoid excessive depletion of NSC pools during on-years can help maintain consistent return bloom and mitigate feedback loops that reinforce AB (Tixier *et al*., 2019). Similarly, rootstocks with enhanced nutrient and water uptake efficiency under limiting conditions have been shown to influence floral induction patterns and yield consistency (Mohammed and Hama Salih, 2024).

A further dimension to consider is the ability of the rootstock to modulate the rhizosphere microbiome, which plays an increasingly recognized role in plant hormonal signaling, stress resilience, and resource acquisition. In *Vitis*, several studies have demonstrated that rootstocks can significantly influence both the composition and the functional profile of root-associated microbial communities, with notable effects on scion physiology, particularly in relation to environmental responsiveness and developmental regulation (Vink *et al*., 2021; Marasco *et al*., 2022). Microbiome-associated traits, although still underexplored in the context of AB, may influence auxin and ABA availability via microbial biosynthesis and degradation pathways, and may also enhance P and N availability via microbial mineralization and solubilization processes (Bhattacharyya and Jha, 2012). These effects could indirectly support floral transition and carbohydrate replenishment, especially under stress-prone conditions.

Looking forward, the identification of rootstock genotypes that can buffer AB under real-world environmental fluctuations will require the integration of long-term field trials with high-resolution physiological and molecular profiling. Combining transcriptomic and epigenomic data with phenotypic traits such as bud fate, NSC dynamics, and nutrient allocation will improve the predictive capacity of selection frameworks. Additionally, root-associated microbial signatures may serve as complementary indicators of rootstock performance under alternating load conditions and environmental stress.

As perennial fruit systems adapt to an increasingly unpredictable climate, the ability of rootstocks to coordinate metabolic stability, hormonal balance, and microbial interactions will be essential to maintain productive capacity and yield consistency. Elucidating the multi-layered signaling processes that operate at the rootstock–scion–soil interface remains a critical priority for AB-oriented breeding strategies, especially as new insights challenge long-standing assumptions about mobile signal identity and call for more rigorous validation of systemic communication pathways (Paajanen *et al*., 2025).
